# Hierarchical NiMn-LDH Hollow Spheres as a Promising Pseudocapacitive Electrode for Supercapacitor Application

**DOI:** 10.3390/mi14020487

**Published:** 2023-02-19

**Authors:** Jai Kumar, Rana R. Neiber, Zaheer Abbas, Razium Ali Soomro, Amal BaQais, Mohammed A. Amin, Zeinhom M. El-Bahy

**Affiliations:** 1 State Key Laboratory of Organic-Inorganic Composites, Beijing Key Laboratory of Electrochemical Process and Technology for Materials, Beijing University of Chemical Technology, Beijing 100029, China; jaik8912@gmail.com; 2 College of Chemical Engineering, University of Chinese Academy of Sciences, 19A Yuquan Road, Beijing 100049, China; rana@ipe.ac.cn; 3 Beijing Key Laboratory of Ionic Liquids Clean Process, CAS Key Laboratory of Green, Process, and Engi-neering, Institute of Process Engineering, Chinese Academy of Sciences, Beijing 100190, China; 4 Metallurgy and Materials Engineering Department, University of Engineering and Technology, Taxila 47050, Pakistan; zaheer.abbas@uettaxila.edu.pk; 5 Department of Chemistry, College of Science, Princess Nourah Bint Abdulrahman University, P.O. Box 84428, Riyadh 11671, Saudi Arabia; aabaqeis@pnu.edu.sa; 6 Department of Chemistry, College of Science, Taif University, P.O. Box 11099, Taif 21944, Saudi Arabia; mo-hamed@tu.edu.sa; 7 Department of Chemistry, Faculty of Science, Al-Azhar University, Nasr City 11884, Cairo, Egypt; zeinel-bahy@azhar.edu.eg

**Keywords:** ssupercapacitors, hollow spheres, layer double hydroxide

## Abstract

Layered double hydroxides (LDH) are regarded as attractive pseudocapacitive materials due to their impressive capacitive qualities that may be adjustable to their morphological features. However, the layered structure of LDH renders them susceptible to structural aggregation, which inhibits effective electrolyte transport and limits their practical applicability after limited exposure to active areas. Herein, we propose a simple template-free strategy to synthesize hierarchical hollow sphere NiMn-LDH material with high surface area and exposed active as anode material for supercapacitor application. The template-free approach enables the natural nucleation of Ni-Mn ions resulting in thin sheets that self-assemble into a hollow sphere, offering expended interlayer spaces and abundant redox-active active sites. The optimal NiMn-LDH-12 achieved a specific capacitance of 1010.4 F g^−1^ at a current density of 0.2 A g^−1^ with capacitance retention of 70% at 5 A g^−1^ after 5000 cycles with lower charge transfer impedance. When configured into an asymmetric supercapacitors (ASC) device as NiMn-LDH//AC, the material realized a specific capacitance of 192.4 F g^−1^ at a current density of 0.2 A g^−1^ with a good energy density of 47.9 Wh kg^−1^ and a power density of 196.8 W kg^−1^. The proposed morphological-tuning route is promising for designing template-free NiMn-LDHs spheres with practical pseudocapacitive characteristics.

## 1. Introduction

Recently, energy calamity has increased concerning the depletion of fossil resources, and thus the development of new technology has received great importance [1]. Currently, energy storage devices, particularly batteries, fuel cells and supercapacitors, are emerging technologies to address energy issues. In particular, supercapacitors are gaining immense attention as auxiliary power sources based on their extended lifetime and high power density [2]. The conventional carbon-based material stores energy via an electric double-layer (EDLC) capacitance mechanism but still requires a lot of work to satisfy the wide-spectrum application of supercapacitors [3,4,5]. On the contrary, the pseudocapacitive material that stores charge via a faradic redox reaction offers greater advantages of tuneable capacitive characteristics with modulation of redox centers or morphology [6,7]. A supercapacitor with two distinct electrode materials is an asymmetric supercapacitor (ASC). One electrode relies on redox (Faradic) reactions with or without non-Faradic reactions, whereas the other relies mostly on non-Faradic or electrostatic double-layer absorption/disproportion. These ASC devices provide a win-win situation in which the combination of typical battery and supercapacitor properties enables the shared aim of better energy density and power density to be attained. In general, the working voltage window for a symmetric supercapacitor is limited to 1.0 V owing to the thermodynamic breakdown of the water molecules within an aqueous electrolyte [6]. However, the utilization of organic electrolytes could expand the working voltage window (>2.5 V), but the toxicity of such electrolytes is an environmental concern. Thus, the use of two different electrodes, i.e., ASC as anode and cathode, is a feasible route to achieve a higher voltage window within an aqueous electrode and generate higher energy and power density [7].

In most common cases, carbon materials are used as a negative electrode, while either a metal oxide or conducting polymer is used as a positive electrode. Here, activated carbon (AC) and graphene have also been used as the negative electrodes of asymmetric supercapacitors because of their robust properties, such as high stability in the negative potential region, good electronic conductivity, large surface area and relatively low cost [8,9]. On the other hand, various transition metal oxides (Mos) and conductive polymers have also been used as positive electrodes because of their fast and reversible electron-exchange reactions at the electrode interface that enable high-power densities and high capacitance. For example, AC/NiO asymmetric supercapacitor has a performing working voltage of 1.5 V with a specific capacitance of 37 F/g [10]. Aqueous AC/MnO_2_ has an operational voltage of 2.2 V with an energy density of 36 Wh/kg [11]. Similarly, AC/V_2_O_5_·0.6H_2_O electrode demonstrated a working voltage region of 0–1.8 V with an energy density of 20.3 Wh/kg [12]. Among the many pseudocapacitive materials, layered double hydroxides (LDHs), with their higher surface area and tuneable layered structure, have become widely known for their impressive capacitive characteristics. The general molecular structure of LDHs can be described as [M_1−x_^2+^ M_x_^3+^ (OH)_2_][ A_x/n_ n^−^·mH_2_O], where M^2+^ and M^3+^ represent the divalent cations and trivalent metallic cations, and A_n_^−^ represents inorganic anions that located in the interlayer [13]. The LDH material, with its positively charged layered surface and hydrated anions in its interlayer, can exhibit capacitive characteristics owing to the redox reaction and shutting of hydroxyl ions during charge-discharge cycles. Thus, the capacitive characteristics of LDH material can be fine-tuned by modulating the layered structures and population of redox-active site centers.

A different combination of metal-LDH, such as NiAl-LDH [14], NiMn-LDH [15], CoAl-LDH [16], and NiCo-LDH [17] has been explored as excellent electrode materials for supercapacitors application. Among different binary metal combinations, the metallic combination of Ni and Mn elements in a layered hydroxide configuration has proven to offer superior redox properties compared to other binary metal counterparts [18]. For example, Zhao and coauthors decorated NiMn-LDH over carbon nanotubes as flexible supercapacitors [19], which exhibited robust pseudocapacitive properties. Sim et al. prepared colloidal NiMn-LDH nanosheets as high-performance supercapacitors electrodes [20].

Though LDHs are gaining their ground in energy storage, layer-stacking is a fundamental problem that limits their practical applicability. This layer-stacking not only minimizes the active site exposure but limits the electrolytic diffusion and surface-redox conversion reaction that eventually compromises the charge-storage and rate performance of the supercapacitor. Several strategies have been suggested, including coupling with carbon-based materials, hybrid composites, and structural modification approaches, to address this bottleneck issue in LDHs. Here, the structural modulation to transform the stacked layered configuration into Hierarchical 3D structures has been proven effective [7,8]. For example, Wan and co-authors proposed an in-situ method to prepare a novel three-dimensional NiCo_2_S_4_@NiMn-LDH@GO architecture as a promising anode for supercapacitors application with high specific capacitance (*C*_sp_) of 1740 mF cm^–2^ at 1 mA cm^–2^ [21]. Furthermore, Zhao et al. [22] prepared NiCo-LDH hollow spheres as a promising active electrode for supercapacitor application. Here, the hollow sphere-like arrangement could offer dual surface area, i.e., interior and exterior, in addition to solving the layer-stacking problem [23,24].

Li and co-authors synthesized hollow spherical morphology like Ni-Mn LDHs with the help of SiO_2_ microspheres as templates [25]. They found that as-prepared hollow microspheres LDH material delivered an excellent capacitive performance with low resistance, and discharge capacitance reached 595.6 F g^−1^ at 1 A g^−1^. Similarly, Jiang et al. investigated different metal combination types of LDH with hollow structures [26]. Here, ZIF-67 nanocrystals were used as templates to produce a hollow cavity. Due to hollow spare-shaped structures, all the prepared LDH materials exhibited superior electrochemical properties. Similarly, ZIF-67 precursors have also been used as a template for hollow NiCo-LDHs–MnO_2_ nanowires @carbon substrates [27,28]. However, the use of the template and its removal could increase the electrode’s fabrication cost. In addition, using a template to build hollow structures considerably complicates the synthesis approach by adding the step of their removal, which may also interfere with the inherent electrochemical characteristics of the material. Therefore, template-free modification of LDH material and its utilization as high-performance pseudocapacitive material is an urgent need for promoting cost-effective energy storage technology. Nevertheless, searching for the optimal structural arrangement to improve capacitive properties is still ongoing. Thus, a simple synthesis strategy that could allow the fabrication of hierarchical hollow sphere NiMn-LDH structures without the assistance of any sacrificial template or seed template would be a viable structure modulation route to produce inherently improved LDHs materials.

Herein, a simple template-free hydrothermal route is proposed to prepare hierarchical hollow spheres of NiMn-LDH for their application as anode material for advanced supercapacitors. Slow nucleation permits the spontaneous self-assembly of Ni and Mn ions into a sheet-like shape, which then adopts a hollow cavity-like morphology. The hollow sphere of NiMn-LDH offers an enlarged layered spacing with abundant redox-active surface sites, whereas the structural arrangement successfully limits the layer stacking, allowing for improved charge-transfer kinetics and pseudocapacitive charge-storage characteristics. The hollow NiMn-LDHs, when configured into an electrode system, could realize a high specific capacitance of 1010.4 F g^−1^ at a current density of 0.2 A g^−1^ with capacitance retention of 70% for 5000 cycles at 5 A g^−1^. When devised into an asymmetric supercapacitor (ASCs) against a carbon counter electrode, the hollow-NiMn-LDH material exhibited a specific capacitance of 192 F g^−1^ at a current density of 0.2 A g^−1^ with a high energy density of 47.9 Wh kg^−1^ at a power density of 196.8 W kg^−1^. The proposed material modification route adds to the progress of template-free methods for producing energy storage materials with promising capacitive characteristics for practical energy storage applications.

## 2. Materials and Methods

### 2.1. Materials

Nickel nitrate hexahydrate (Ni (NO_3_)_2_·6H_2_O), manganese chloride tetrahydrate (MnCl_2_·4H_2_O), and methanol (CH_3_OH) were obtained from Sigma Aldrich chemical with high analytical purity of more than 99%.

### 2.2. Synthesis of Hierarchical Hollow-NiMn LDHs

The hierarchical hollow sphere NiMn-LDHs were synthesized using a simple template-free hydrothermal route. In a typical experiment, a 5:25 specific ratio of H_2_O: CH_3_OH was initially homogenized for 15 min. The mixture was gradually introduced with a 1:1 molar ratio of Ni and Mn solution followed by 1 h sonication. The mixture was later placed in a 50 mL autoclave reactor and hydrothermally treated at 180 °C for 12 h. The precipitates were subsequently washed with deionized water and dried at 60 °C for 24 h before configuring into an anode for the supercapacitor. Ni and Mn ratios in hollow sphere NiMn-LDHs were optimized for best performance, with ratios of 1:1, 1:2, 1:3, and 1:5 denoted as NiMn-LDH-11, NiMn-LDH-12, NiMn-LDH-13, and NiMn-LDH-15, respectively.

### 2.3. Characterizations

Hollow sphere NiMn-LDH was characterized using advanced analysis techniques such as HR-SEM (JSM-6701F (JEOL, Japan), which was used to study the morphological features of hollow NiMn LDH with energy-dispersive X-ray spectroscopy (EDX) for elemental composition. X-ray diffraction (XRD) (Cu Ka, k = 1.5406 Å; Rigaku Corporation, Tokyo, Japan) and X-ray photoelectron spectroscopy (XPS, VG Scientific ESCALAB 250) were used for compositional characteristics. XPS data were fitted using Casa XPS software version 2.31.

#### Electrochemical Analysis

The working electrode was constructed using 80% NiMn LDHs as active material, 10% acetylene black as conductive material and 10% PTEF as a binder. The electrochemical assessment was carried out in a T-cell electrode with NiMn-LDH as the working electrode, activated carbon as the counter electrode, and Ag/AgCl as a reference electrode with 3.0 M KOH as an electrolyte, respectively. The mass of the counter electrode was taken 3.5 times of working electrode after balancing the electrode charges using the following equation:

(1) $$\frac{m^{+}}{m^{-}}=\frac{C^{-}\times \Delta V^{-}}{C^{+}\times \Delta V^{+}}$$

where m represents the mass of the electrode in (g), C is the specific capacity (F/g), and ∆V represents the potential range (V).

The assembled electrode was characterized by using efficient techniques such as cyclic voltammetry (CV), galvanostatic charge-discharge (GCD) and electrochemical impedance spectroscopy (EIS). The storage-specific capacitance of NiMn-LDH materials was calculated from CV and GCD according to Equations (2) and (3) [29], respectively.

(2) $$C=\frac{1}{\mathrm{mk}\;\left(V_{2}-V_{1}\right)}\int_{V_{1}}^{V_{2}}i\;\left(V\right)\mathrm{dV}$$

(3) $$C=\frac{i\times \Delta t}{m\;\times \Delta V}$$

where V_1_ and V_2_ represent the lower and upper limits of potential (V), respectively. i(V) is the current(A), dV is the potential differential (V), k is the potential scan rate (mVs^−1^), and m is the mass of electroactive material (g).

Asymmetric supercapacitors (ASCs) NiMn-LDH//AC were assembled using NiMn-LDH as the positive electrode and active carbon (AC) as a negative electrode, respectively, while CV and GCD were also produced using two system configurations.

## 3. Results and Discussion

### 3.1. Synthesis and Characterization of Hollow Sphere NiMn-LDH

**Figure 1:**
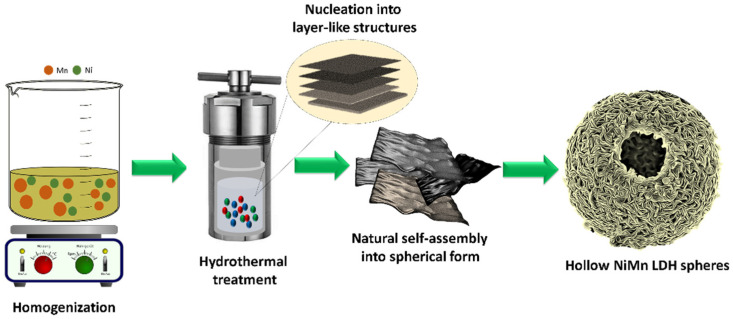
Schematic illustration of NiMn-LDH hollow sphere preparation by hydrothermal treatment.

Various extrinsic and intrinsic properties are responsible for the final morphology of the products. In general, crystal growth and morphology are rooted in the degree of supersaturation, the species around the surface of the crystals, and the interfacial energies. Our approach here is based on the molecular chemistry of metal oxides in which the emulsion plays an important role in preferential growth. A simple template-free route was adopted to prepare hierarchical hollow NiMn LDH spheres (Figure 1), where slow nucleation of Ni and Mn enabled its layered structure following the formation of hollow-sphere-like morphology.

**Figure 2:**
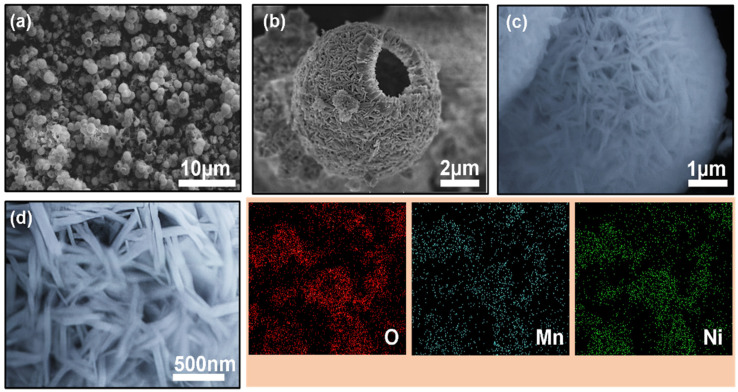
(**a**–**c**) HR-SEM images of NiMn-LDH-12 hollow sphere at different magnifications with corresponding (**d**) SEM-EDX image confirming the uniform distribution of O (oxygen), Mn (manganese), and Ni (nickel) elements.

HR-SEM was used to assess the morphology of the prepared hollow sphere NiMn-LDHs. Figure 2a–c shows representative HR-SEM images of NiMn-LDH-12. As seen, the NiMn LDH adopted a hollow-cavity containing morphology composed of thin layers arranged in spherical form (Figure 2c) with average flake thickness in the range of 50 to 90 nm ± 3.5 nm. Figure 2d shows the SEM image and corresponding elemental mapping confirming the uniformly distributed Ni, Mn and O elements in NiMn-LDH-12. Unlike the typical layered structure of NiMn LDH, the hollow spheres provide enlarged layered spacing with abundant active sites and a loose structural configuration that offers limited aggregation between the layers reflecting its ability to facilitate the charge-transfer process and eventually rate the performance of the electrode.

**Figure 3:**
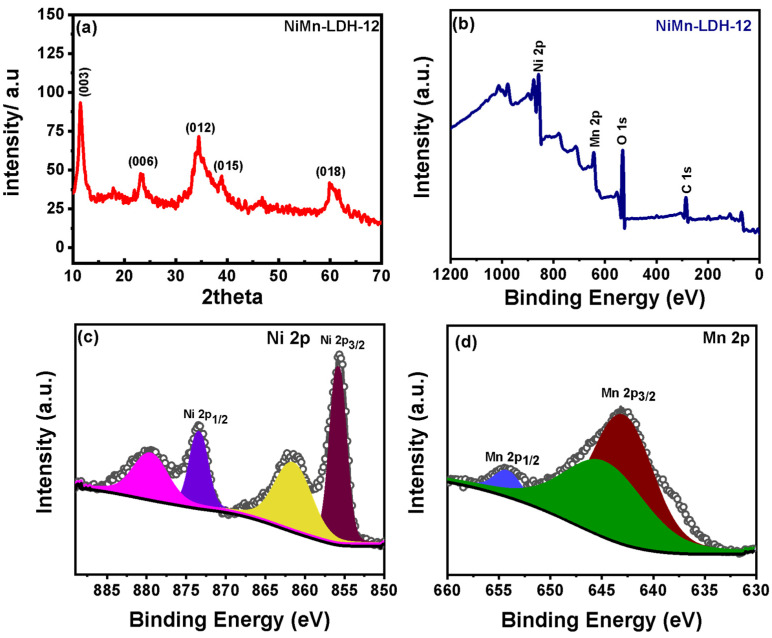
(**a**) X-ray diffraction pattern of the representative NiMn-LDH-12 hollow sphere; (**b**) corresponding XPS survey spectrum; (**c**) the high-resolution Ni 2p and; (**d**) Mn 2p binding energies.

Figure 3a shows the XRD pattern of representative NiMn-LDH-12 with typical peaks at 11.9°, 23.2°, 34.48°, 38.92° and 59.9° which can be indexed to the (003), (006), (012), (015) and (018) planes of NiMn LDH as referenced against ICCD card number: JCPDS 38-0715 [30]. The absence of any irrelevant peak further confirms the analytical reliability of the proposed route and the chemical purity of the hollow spheres. The chemical state of the NiMn-LDH-12 was evaluated using XPS. Figure 3b shows the survey spectrum with typical peaks of Ni, Mn, and O, confirming the pristine chemical composition of the material. The high-resolution spectra (Figure 3c) of Ni were further deconvoluted for Ni 2p peaks at 855.8 eV and 873.3 eV, which correspond to Ni 2p_3/2_ and Ni 2p_1/2_ with satellite peaks at 861.6 eV and 879.6 eV respectively [31]. The high-resolution spectra for Mn 2p binding energy (Figure 3d) could be fitted for three distinct peaks at 643.164 eV and 654.3 eV, which correspond to Mn 2p_3/2_ and Mn 2p_1/2_, and a satellite peak at 645.5 eV [21]. In addition, the O1s spectrum (Figure S1) was fitted with two peaks at 531.36 eV and 531.8 eV that are associated with metal–oxygen–metal and metal–oxygen–hydrogen bonds from the M(OH)_6_ (M denoted: Ni or Mn) and a small peak at 531.06 eV that was associated with hydrogen–oxygen–hydrogen binding [32]. The results revealed the valence states of Ni and Mn were divalent and trivalent in the LDH configuration.

### 3.2. Electrochemical Performance

**Figure 4:**
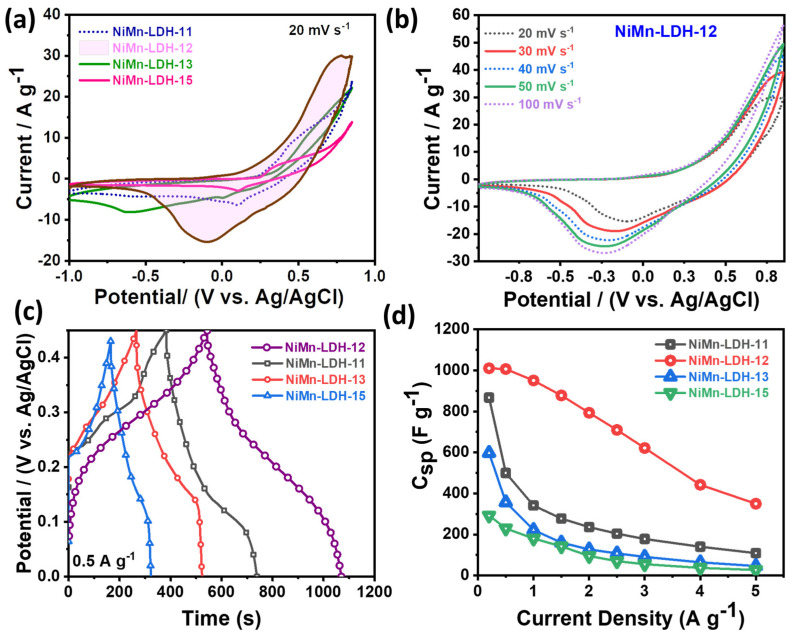
(**a**) CV profile of NiMn-LDH-12 in comparison to its other compositional counterparts; (**b**) corresponding peak current variation against different scan rates in the range of 20 to 100 mV s^−1^ in a fixed potential window of −1 to 0.85 V; (**c**) Charge discharge curves for NiMn-LDH-11, 12, 13 and 15 at a constant current density of 0.5 A g^−1^. (**d**) comparison of specific capacitance of NiMn-LDHs with different Ni: Mn ratios at different current densities under a potential window of 0 to 0.45 V.

The electrochemical analysis was evaluated using cycling voltammetry (CV) and galvanostatic charge/discharge measurements. Figure 4a shows the CV curves for NiMn LDH materials with different Ni: Mn ratios of 1:1 to 1:5, with CV recorded at 20 mVs^−1^. The CV profiles of NiMn LDHs (i.e., NiMn LDH-11, 12, 13, and 155) consisted of typical redox peaks that were attributed to the Ni (II/III) and Mn (II/III) conversions, confirming the faradic behavior of the material. The typical peaks could be ascribed to the reversible reduction and oxidation reaction of Ni^2+^/Ni^3+^ and Mn^2+^/Mn^3+^ in an alkaline solution (3M, KOH) represented by the following chemical equation [33]:

Ni(OH)_2_–LDH + OH^−^ ↔ NiOOH–LDH + H_2_O + e^−^ (4)

Mn(OH)_2_–LDH + OH^−^ ↔ MnOOH–LDH + H_2_O + e^−^ (5)

MnOOH–LDH + OH^−^ ↔ MnO_2_–LDH + H_2_O + e^−^ (6)

As seen in Figure 4a Ni: Mn ratio of 1:2 (NiMn-LDH-12) realized a high current density and much broader capacitive region confirming the superior synergism of Ni and Mn at equal counterparts compared to NiMn LDHs-11, 13, and 15. To confirm the charge-transfer characteristics, CV profiles were recorded at different scan rates in the range from 20 to 100 mV s^−1^. In the case of NiMn-LDH-11 and 13 (Figure S2a,b), the redox peaks could be seen gradually diminishing with increasing scan rate confirming its poor charge-transfer performance. The redox peaks in the case of NiMn-LDH-15 (Figure S2c) could be seen maintained at higher scan rates. However, the overall current density and the capacitive region were much smaller than NiMn-LDH-12 (Figure 4b). Thus, the NiMn-LDH-12 was considered an optimal material for device construction and capacitive assessment.

The galvanotactic charge-discharge (GCD) measurement was carried out for NiMn-LDH-11, NiMn-LDH-12, NiMn-LDH-13, and NiMn-LDH-15. The corresponding GCD profiles at a fixed current density of 0.5 A g^−1^ are shown in Figure 4c. As expected, the NiMn-LDH-12 delivered the highest discharge rate and nonlinear charge-discharge profile, which agrees with its CV profile, confirming the pseudocapacitive nature. Figure S3a–d shows the GCD profiles of the materials at different current densities ranging from 0.5 A g^−1^ to 5 A g^−1^, where the typical non-isosceles triangular charge-discharge profiles indicate the faradic behavior related to double-layer capacitance and pseudo capacitance. The GCD profile at varied current densities exhibits similar non-linear behavior with a continuously declining discharge rate by reducing the current density. Figure 4d shows the specific capacitance of the as-prepared three samples at different current densities estimated from their charge-discharge profile. The determined specific capacitance indicates that NiMn-LDH-12 has the superior storing capability to control samples. The specific capacitance of NiMn-LDH-11, NiMn-LDH-12, NiMn-LDH-13 and NiMn-LDH-15 was calculated to be 867.19, 1010.4, 597.5, 292.2 F g^−1^ at 0.2 A g^−1^, 499.7, 1006.4, 357.1, 229.38 F g^−1^ at 0.5 A g^−1^, and 108.8, 349.1, 46.1, 26.9 F g^−1^ at 5 A g^−1^, respectively. Figure S4 depicts the specific capacitance of the as-prepared NiMn-LDH-12 derived from typical CV curves at various scan rates, which likewise exhibit a promising capacitive behavior. The obtained corresponding specific capacitance shows NiMn-LDH-12 hollow sphere possesses higher specific capacitance even at lower current density than NiMn-LDH-11, NiMn-LDH-13 and NiMn-LDH-15, demonstrating hollow NiMn-LDH-12 sphere promising capability for supercapacitor application. A comparison between the as-prepared NiMn-LDH-12 hollow sphere with other reported Ni and Mn-based materials is tabulated in Table 1, showing the acceptable performance of the hollow sphere NiMn-LDH-12 for energy application.

The CV profiles were recorded at different scan rates in the range from 10 to 100 mV s^−1^. The following power-law equation was applied to estimate the charge storage kinetics of the NiMn-LDH-12:

i = a*v*^b^ (7)

where i and *v* symbolize peak current and scan rate, while a and b are variables. The b value could be obtained from the slope of the graph between log (*v*) vs. log (i). Notably, a “b” value of 0.5 designates a diffusion-controlled process, while a “b” value of 1 designates a non-diffusion controlled capacitive process.

**Figure 5:**
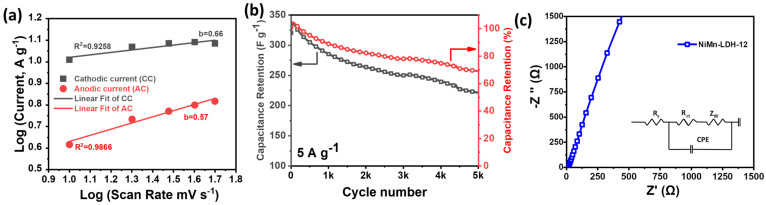
(**a**) The plot of anodic and cathodic peak current responses of NiMn-LDH-12 against the log of different scan rates with a linear fit analysis; (**b**) The cycling stability of NiMn-LDH-12 at a constant current density of 5 A g^−1^ with inset showing representative charge-discharge curve after fixed intervals; (**c**) EIS based Nyquist plot for hollow sphere NiMn-LDH-12.

Figure 4b shows the gradually changing CV curves of NiMn-LDH-12 with increasing scan rates. The plot of peak current *v. s.* scan rates estimated a “b” value of 0.66 for a cathodic sweep and 0.57 for the anodic sweep, signifying the predominance of a diffusion-controlled process (Figure 5a).

The stability of the hollow sphere NiMn-LDH-12 was evaluated for the 5000 continuous charge-discharge cycles at 5 A g^−1^ current density. Figure 5b shows that the NiMn-LDH-12 hollow sphere has a near-stable cycle performance with capacitive retention of around 70% after 5000 cycles.

The performance of NiMn-LDH-12 could be attributed to the overall structural configuration where the hollow-structural features allow minimum layer-stacking and improved surface area to realize larger interlayer spacings with greater active sites and superior conductive, as supported by the small semi-circle in the low high-frequency and a straight line in high-frequency region of the EIS based Nyquist plots (Figure 5c).

**Figure 6:**
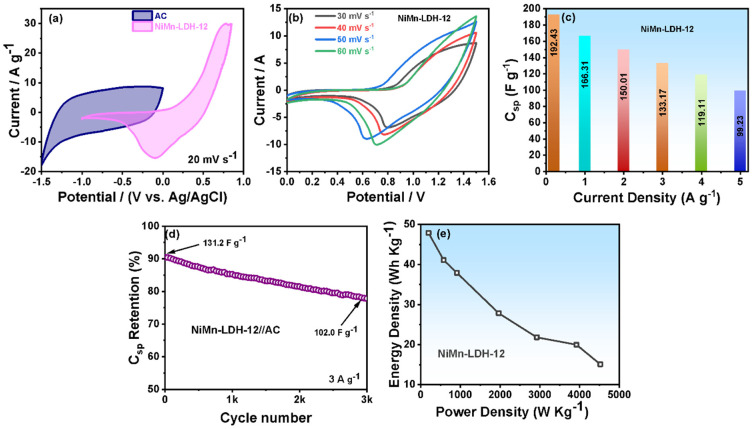
(**a**) CV curve of AC and NiMn-LDH-12 with overlapping potential window; (**b**) CV profile of NiMn-LDH-12//AC at different scan rates in a fixed potential window of 0 to 1.5 V; (**c**) specific capacitance of resulting ASC device at different current densities; (**d**) cycling stability measurement at 3000 cycles at constant current density under the potential window of 0 to 1.5 V and; (**e**) the plot of energy and power density of the constructed ASC device.

To find the practical application of NiMn-LDH-12 material, an asymmetric supercapacitor was engineered with activated carbon (AC) as a counter electrode (NiMn-LDH-12//AC). Figure 6a shows the typical CV measurement of NiMn-LDH-12 and AC in 3 M KOH and a potential window of −1.5 to 0 V with the symmetric rectangular curve of AC showing EDLC behavior, while NiMn-LDH-12 material revealed a faradic behavior in the potential range of 0 to 0.85 V. The CV profile of NiMn-LDH-12//AC was recorded at different scan rates ranging from 30 mV s^−1^ to 60 mV s^−1^ (Figure 6b), showing a stable pair of reduction and oxidation peaks in alkaline (3 M KOH) electrolyte. Furthermore, the capacitance was calculated to be 192.43 F g^−1^ at 0.2 A g^−1^ with a retained capacitance of 99.23 F g^−1^ at 5 A g^−1^ (Figure 6c).

The long-term cycling stability of the NiMn-LDH-12//AC was determined for 3000 cycles at 3 A g^−1^. As shown in Figure 6d, the ASC device could be maintained around 77.6% after the 3000th cycle to its initial performance. Furthermore, the energy and power density of the ASC device was calculated according to the given equation:

(8) $$E\;=\frac{1}{2}\times C\times {\left(△V\right)}^{2}$$

(9) $$P\;=\frac{E}{△T}$$

Figure 6e shows the calculated energy and power density of the ASC device. The energy density of the ASC device reached its highest value of around 47.9 Wh kg^−1^ at a power density of 196.8 W kg^−1^, which is considerably greater than other composite materials such as ZnNiCoO_4_@CoWO_4_//AC (7.4 Wh kg^−1^ at a power density of 181.2 W kg^−1^) [37], V_2_O_5_/rGO//AC (7.4 Wh kg^−1^ at a power density of 127.2 W kg^−1^) [48], CoGa_2_O_4_@CC||AC@CC (0.9 Wh kg^−1^ at a power density of 13.4 W kg^−1^) [49] and Ni-Co-Cu-oxide//AC (0.7 Wh kg^−1^ at a power density of 13.8 W kg^−1^) [50].

## 4. Conclusions

A simple and effective template-free hydrothermal route was proposed to synthesize hierarchical hollow sphere NiMn LDHs. The proposed strategy enabled the formation of exaggerated structures with minimum layer-stacking and abundant active surface sites owing to expended interlayer surface features of hierarchical hollow sphere NiMn LDHs. To evaluate the electrochemical qualities, several Ni: Mn ratios in the range of 1:1, 1:2, 1:3, and 1:5 was investigated, where the representative NiMn LDH-12 exhibited superior electrochemical performance compared to its other compositional counterparts. The NiMn LDH-12, when configured as an anode for a supercapacitor, revealed a maximum specific capacitance of 1010.4 F g^−1^ at a current density of 0.2 A g^−1^ with capacitance retention of 70% at 5 A g^−1^ after 5k cycles. Moreover, an asymmetric supercapacitors (ASC) device assembled as NiMn-LDH-12//AC exhibited a specific capacitance of 192.4 F g^−1^ at a current density of 0.2 A g^−1^ with a high energy density of 47.9 Wh kg^−1^ at a power density of 196.8 W kg^−1^. The suggested strategy provides new insight into the advancement of template-free strategies for producing energy storage materials with promising capacitive properties.

## Figures and Tables

**Table 1:** Analytical comparison of NiMn-LDH-12 with similar reported materials.

Composite Material	Specific Capacitance (F g^−1^)	Current Density (A g^−1^)	Electrolyte	Ref.
MnCo_2_O_4.5/_NiCo_2_O_4_	325	1	3 M KOH	[34]
CoWO_4_–Ni_3_	271	1	6 M KOH	[35]
MnMoO_4_/CoMoO_4_	187	1	2 M NaOH	[36]
ZnNnCO_4_/CWO_4_	309	1	2 M KOH	[37]
Hollow shelled Mn–Cu–Al–oxide	319	1	1 M Na_2_SO_4_	[38]
Core-shell CuCo_2_O_4_/MnO_2_ nanowires	327	1.25	3 M KOH	[39]
Starfish-shaped Co_3_O_4_/ZnFe_2_O_4_ nanocomposite	326	1	6 M KOH	[40]
Co–Al LDH/graphene	712	1	6 M KOH	[41]
Co–Al LDH/Pt	734	3	2 M KOH	[42]
Co–Al LDH/graphene	772	1	6 M KOH	[43]
CoAl-LDH/PEDOT	672	1	6 M KOH	[44]
NiAl LDH	795	0.5	1 M KOH	[45]
NiAl LDH	735	2	1 M KOH	[46]
CoAl LDH/graphene	479	1	6 M KOH	[47]
Hollow sphere NiMn-LDH-12	951	1	3 M KOH	This Work

## Data Availability

Not applicable.

## Supplementary Materials

The following supporting information can be downloaded at: https://www.mdpi.com/2072-666X/14/2/487/s1. Figure S1: XPS fitting profile of O1s of NiMn-LDH-12 material; Figure S2: CV profile of (a) NiMn-LDH-11, (b) NiMn-LDH-13 and (c) NiMn-LDH-15 at different scan rate; Figure S3: GCD profile of (a) NiMn-LDH-11, (b)NiMn-LDH-12, (c) NiMn-LDH-13 and (d) NiMn-LDH-14 at the different scan rate. Figure S4: Specific capacitance profile calculated from the CV plots of NiMn-LDH-12 at different scan rates.
