# Supplementary material for: Hierarchical NiMn-LDH Hollow Spheres as a Promising Pseudocapacitive Electrode for Supercapacitor Application
Source: Micromachines (Basel). 2023 Feb 19;14(2):487. doi: 10.3390/mi14020487 (PMC9964479; doi:10.3390/mi14020487)
Supplement: Supplementary file 1 [file micromachines-14-00487-s001.zip › micromachines-2207990-supplementary.pdf]

Article

Hierarchical NiMn-LDH hollow spheres as a promising pseudocapacitive electrode for hybrid type supercapacitor application

Jai Kumar[1], Rana R. Neiber[1, 2], Zaheer Abbas[3], Razium Ali Soomro[4, *], Amal BaQais[5], Mohammed A. Amin[6], Zeinhom M. El-Bahy[7]

1 College of Chemical Engineering, University of Chinese Academy of Sciences, 19 A Yuquan Road, Beijing, 100049, China
2 Beijing Key Laboratory of Ionic Liquids Clean Process, CAS Key Laboratory of Green, Process, and Engineering, Institute of Process Engineering, Chinese Academy of Sciences, 100190 Beijing, China
3 Metallurgy and Materials Engineering Department, University of Engineering and Technology, Taxila, Pakistan
4 State Key Laboratory of Organic-Inorganic Composites, Beijing Key Laboratory of Electrochemical Process and Technology for Materials, Beijing University of Chemical Technology, Beijing 100029, China
5 Department of chemistry, college of science, Princess Nourah Bint Abdulrahman university, P.O. Box 84428, Riyadh 11671, Saudi Arabia
6 Department of Chemistry, College of Science, Taif University, P.O. Box 11099, Taif 21944, Saudi Arabia
7 Department of Chemistry, Faculty of Science, Al-Azhar University, Nasr City 11884, Cairo, Egypt

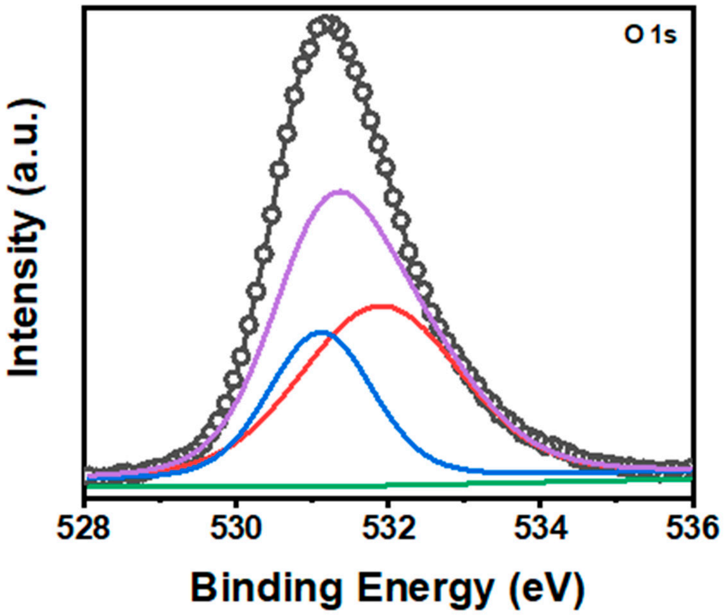

Figure S1. XPS fitting profile of O1s of NiMn-LDH-11.

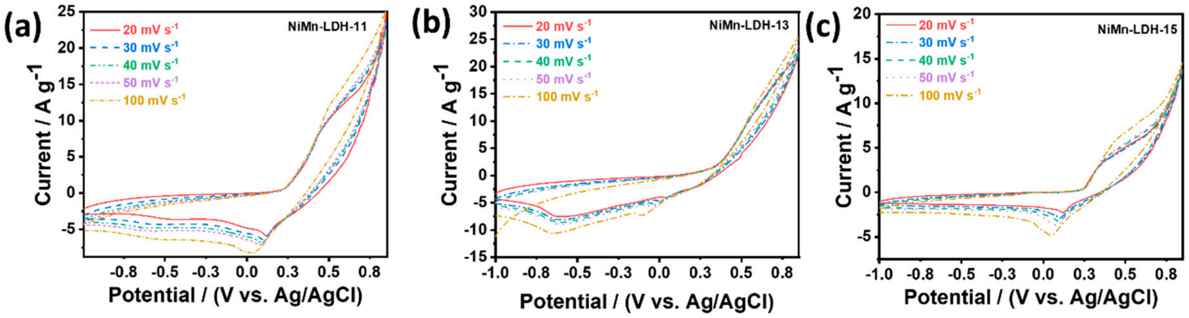

Figure S2. CV profile of (a) NiMn-LDH-13 and (b) NiMn-LDH-15 at different scan rates.

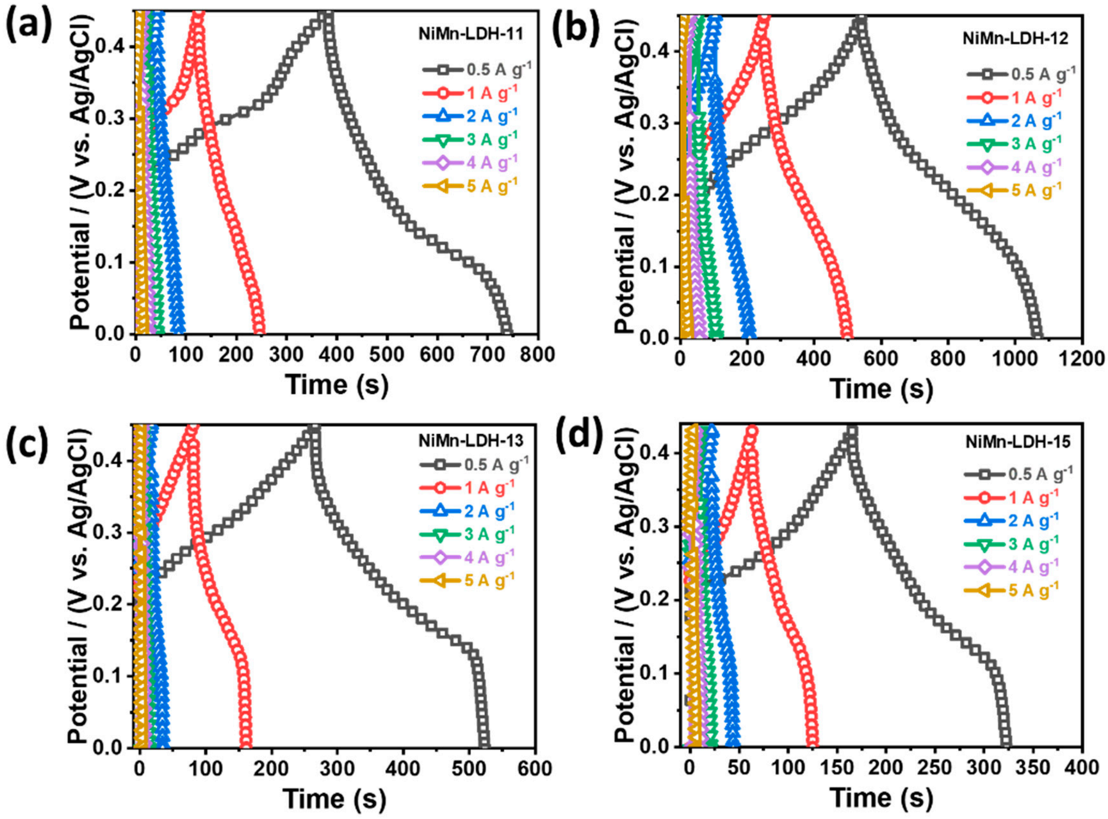

Figure S3. GCD profile of (a) NiMn-LDH-11, (b) NiMn-LDH-13 and (c) NiMn-LDH-13 at different scan rates.

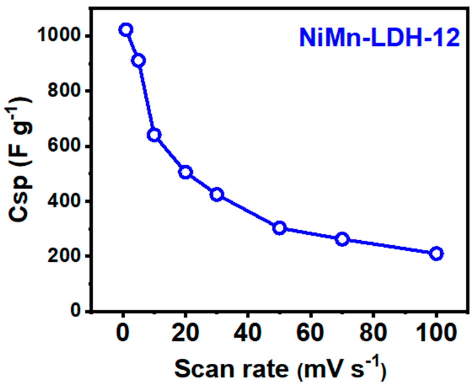

Figure S4. Specific capacitance profile calculated from the CV plots of NiMn-LDH-12 at different scan rates.
